# A dynamical systems model for the total fission rate in Drp1-dependent mitochondrial fission

**DOI:** 10.1371/journal.pcbi.1012596

**Published:** 2024-11-18

**Authors:** Anna K. Leinheiser, Colleen C. Mitchell, Ethan Rooke, Stefan Strack, Chad E. Grueter

**Affiliations:** 1 Department of Mathematics, University of Iowa, Iowa City, Iowa, United States of America; 2 Department of Internal Medicine, Division of Cardiovascular Medicine, Francois M. Abboud Cardiovascular Research Center, Fraternal Order of Eagles Diabetes Research Center, University of Iowa, Iowa City, Iowa, United States of America; 3 Department of Neuroscience and Pharmacology, University of Iowa, Iowa City, Iowa, United States of America; University of Michigan, UNITED STATES OF AMERICA

## Abstract

Mitochondrial hyperfission in response to cellular insult is associated with reduced energy production and programmed cell death. Thus, there is a critical need to understand the molecular mechanisms coordinating and regulating the complex process of mitochondrial fission. We develop a nonlinear dynamical systems model of dynamin related protein one (Drp1)-dependent mitochondrial fission and use it to identify parameters which can regulate the total fission rate (TFR) as a function of time. The TFR defined from a nondimensionalization of the model undergoes a Hopf bifurcation with bifurcation parameter $\mu =\frac{k_{+}M}{k_{-}}$ where M is the total concentration of mitochondrial fission factor (Mff) and *k*_+_ and *k*_−_ are the association and dissociation rate constants between oligomers on the outer mitochondrial membrane. The variable *μ* can be thought of as the maximum build rate over the disassembling rate of oligomers. Though the nondimensionalization of the system results in four dimensionless parameters, we found the TFR and the cumulative total fission (TF) depend strongly on only one, *μ*. Interestingly, the cumulative TF does not monotonically increase as *μ* increases. Instead it increases with *μ* to a certain point and then begins to decrease as *μ* continues to increase. This non-monotone dependence on *μ* suggests interventions targeting *k*_+_, *k*_−_, or M may have a non-intuitive impact on the fission mechanism. Thus understanding the impact of regulatory parameters, such as *μ*, may assist future therapeutic target selection.

## Author summary

Environmental and metabolic stress can lead to a state of mitochondrial hyperfission which is associated with functional defects and multiple human diseases. We construct a mathematical model in order to better understand a cell’s transition to hyperfission. The interaction of the cytosolic protein dynamin related protein one (Drp1) and the mitochondrial membrane receptor mitochondrial fission factor (Mff) is necessary for fission to occur. Thus, we develop a nonlinear dynamical systems model for Drp1-dependent mitochondrial fission which tracks Drp1-Mff complexes from a Drp1 recruitment phase to a fission event. Using numerical simulations, parameter sensitivity analyses, and a bifurcation analysis, we identify the key parameters that influence the total fission rate (TFR) as a function of time. We also discover that the TFR undergoes a Hopf bifurcation and exhibits a non-monotone dependence on certain rate parameters. Hence, the model provides a mathematical framework to understand the molecular mechanisms of Drp1-dependent mitochondrial fission and suggests potential regulatory parameters for modulating fission.

## 1 Introduction

Mitochondria are dynamic organelles that maintain a steady-state through three general processes: mitochondrial biogenesis, mitophagy, and mitochondrial dynamics (fission and fusion) [1–6]. Upon acute stress, mitochondria can undergo hyperfission resulting in an increase in fragmentation, which shifts their size distribution [7]. These fragmented mitochondria have decreased ATP production, decreased oxygen consumption, increased reactive oxygen species production, and an increased susceptibility to programmed cell death [8] [7]. Moreover, inhibition of mitochondrial hyperfission can blunt the impact of cellular injury. In cardiomyocyte-like HL1 cells, through the inhibition of fission signaling (including cyclin dependent kinases Cdk 1, 2 and 5), roscovitine protects cells from ischemia/reperfusion injury in vitro [8]. Further in vivo studies in rats demonstrate that inhibiting hyperfission in early stages of ischemia protect against long-term ischemic injury including a decrease in infarct size and improvement in function [9]. In male mice, studies demonstrate inhibition of Drp1-dependent mitochondrial fission by the outer mitochondrial AKAP1/PKA complex protects neurons from ischemic stroke [10]. However, deletion of key mitochondrial fission and fusion proteins (Mff or Drp1) results in heart failure [11]. Disruptions in the balance between fission and fusion has also been observed to affect normal development, and these disruptions have been implicated in neurodegenerative diseases [12] indicating that a balance between fission and fusion is crucial to cell health across diverse cell types.

Drp1 is a key player in mitochondrial fission through its interaction with the outer mitochondrial membrane (OMM) [13–17]. Drp1 is recruited to the OMM and binds to membrane receptors such as mitochondrial fission factor (Mff), MiD49, and MiD51 [13, 17–20]. The receptor Mff is essential for recruiting Drp1 to mitochondria [17], and over-expression of Mff increases mitochondrial fission while over expression of MiD49 and MiD51 inhibits fission [13, 20]. For these reasons, the nonlinear dynamical systems described below focus on Drp1 and Mff interactions. Drp1 oligomerizes to form a chain like structure, an oligomer, which wraps around the mitochondria leading to a fission event. While Drp1 recruitment, oligomerization, and interaction with outer mitochondrial membrane receptors is known to occur [13, 18, 19], the specific contributions of these processes to the fission rate is incompletely understood.

Therefore there is a need to understand the molecular mechanisms coordinating mitochondrial dynamics. We develop a nonlinear dynamical system for a Drp1-dependent fission mechanism and use parameter sensitivity analyses, bifurcation analysis, and a nondimensionalization of the model to identify which parameters have the greatest effect on the total fission rate (TFR) as a function of time. The TFR is the number of fission events per second. We will refer to our nonlinear dynamical system as the Fission Model. The TFR defined from the nondimensionalization of the Fission Model undergoes a Hopf bifurcation with bifurcation parameter $\mu =\frac{k_{+}M}{k_{-}}$, where M is the total concentration on Mff; *k*_+_ and *k*_−_ are the association and dissociation rate constants, respectively, between oligomers and single Drp1-Mff complexes on the outer mitochondrial membrane. The parameter *μ* can be thought of as the maximum build rate over the disassembling rate of oligomers. Even though the nondimensionalization of the system results in four dimensionless parameters, we found the cumulative total fission (TF), which is the total number of fission events over a set time period, depends strongly on only one of them, *μ*. Thus we identify *μ* to be a key regulatory parameter for Drp1-dependent mitochondrial fission.

## 2 Model: A nonlinear dynamical system for Drp1-dependent mitochondrial fission

**Fig 1:**
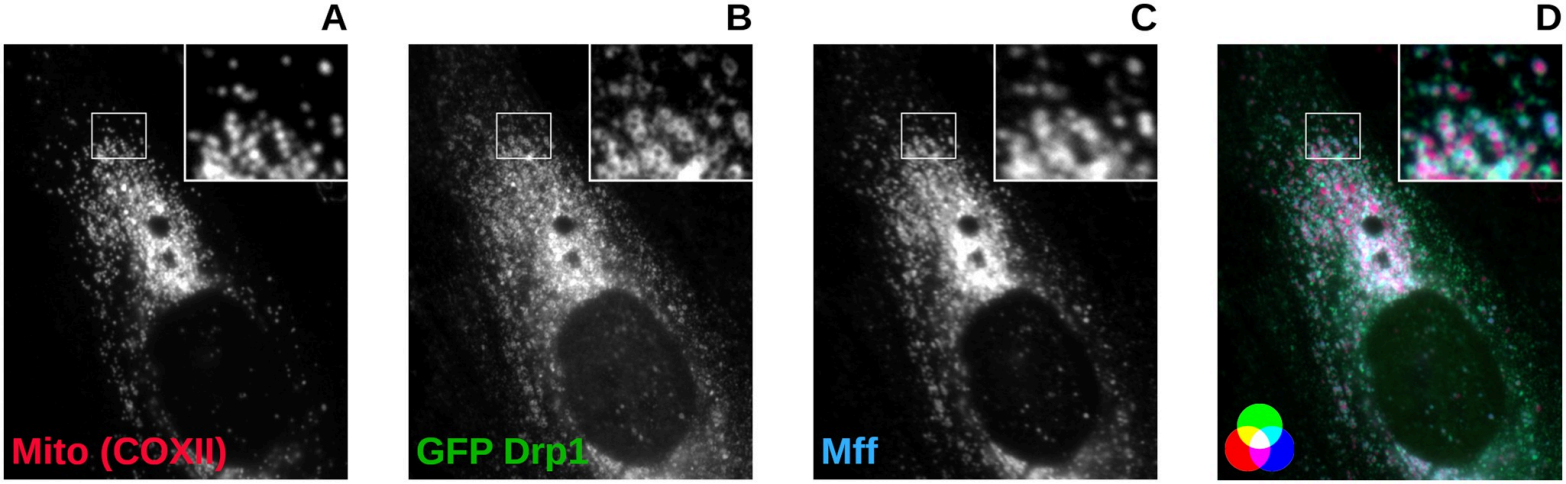
Confocal images of heterologous cells probed for the mitochondrial marker COXII (A), transiently transfected with GFP-Drp1 (B), and stained for Mff (C). The overlayed image (D) shows Drp1/Mff (green/blue) complexes in cyan. Note the cyan ring structures encircling the red mitochondria. This demonstrates Drp1 oligomerization with Mff on mitochondria.

Mitochondrial fission is the process of a mitochondrion dividing into two daughter organelles. The Fission Model presented here is a system of nonlinear ordinary differential equations (ODEs) which focus on Drp1-dependent mitochondrial fission following cellular injury when the concentration of Drp1 in the cytosol is increased. The mechanism our model approximates begins with the recruitment and binding of cytosolic Drp1 tetramers with Mff anchored to the OMM via its hydrophobic C-terminal transmembrane domain. Since Drp1 exists in a predominately tetrameric form in the cytosol [14], the model focuses on the recruitment of Drp1 tetramers. Fig 1B shows GFP tagged Drp1 encircling the mitochondria, which illustrates the recruitment phase of the mechanism. Fig 1D shows red mitochondria encircled with a cyan ring structure where the cyan color is from the overlay of green Drp1 and blue Mff. This illustrates Drp1-Mff interactions on the OMM.

**Fig 2:**
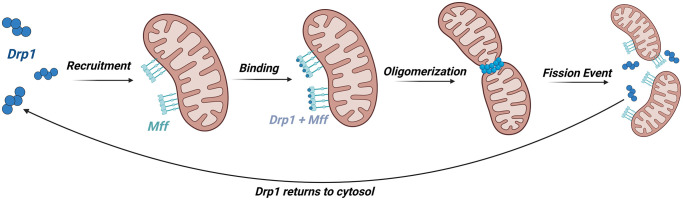
The mechanism our model approximates begins with the recruitment of Drp1 tetramers from the cytosol to the outer mitochondrial membrane where they bind to Mff. These Drp1-Mff complexes build oligomers which constrict around the outer membrane and lead to a fission event. After a fission event, Drp1 tetramers and Mff proteins return to their respective unbound pools in the cytosol and on the outer mitochondrial membrane. Created with BioRender.com.

The model tracks the concentration of these Drp1-Mff complexes as they build oligomers of various sizes. Once an oligomer reaches a sufficient size, which will be discussed in more detail later, a fission event occurs. After a fission event, Drp1 tetramers and Mff proteins instantly return to their respective unbound pools within the model (Fig 2). The Fission Model is a common pool model and represents the fission activity of an entire cell. Therefore, the concentration of Mff on the OMM is the concentration across all mitochondria. The model focuses on the oligomerization process in order to better understand how changes upstream of a fission event affect the number of fission events per second.

The variables and parameters that makeup the Fission Model are

*T*: concentration of cytosolic Drp1 tetramers—units *nM*
*M*: concentration of Mff in an unbound form anchored to the OMM—units *nM*
*C*_1_: concentration of Drp1-Mff complexes (oligomers of size one) on the OMM—units *nM*
*C*_*i*_: concentration of oligomers of size *i* for 2 ≤ *i* ≤ *N* on the OMM—units *nM*
*k*_1_: association rate constant for cytosolic Drp1 and Mff on the OMM—units (*nMs*)^−1^
*k*_−1_: dissociation rate constant of a Drp1 tetramer to unbind from Mff—units *s*^−1^
*k*_+_: association rate constant for Drp1-Mff complexes (*C*_1_) and oligomers *C*_*i*_—units (*nMs*)^−1^
*k*_−_: dissociation rate constant for Drp1-Mff complexes (*C*_1_) and oligomers *C*_*i*_—units *s*^−1^
*f*(*i*): fission rate as a function of oligomer size—units *s*^−1^

The size of an oligomer refers to the number of Drp1-Mff complexes that makeup the oligomer. Oligomers of size *i* ≥ 2 on the OMM are assembled/disassembled one complex at a time, e.g. an oligomer of size four is formed from the association of a *C*_3_ and a *C*_1_ or the dissociation of a *C*_1_ from a *C*_5_. For *i* ≥ 1, the following reaction equation expresses the oligomerization process,

$$\begin{array}{c} C_{i}+C_{1}\underset{k_{-}}{\overset{k_{+}}{⇆}}C_{i+1}. \end{array}$$ (1)

**Fig 3:**
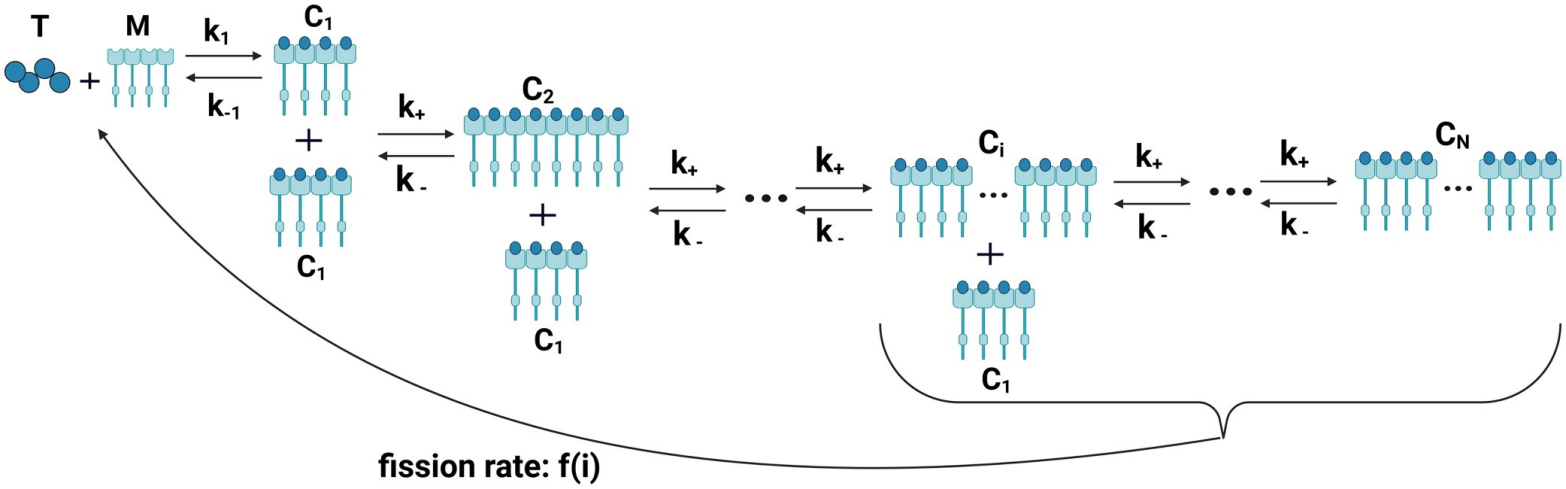
A reaction schematic for the Fission Model: *T*—concentration of unbound Drp1 tetramers in the cytosol. *M*—concentration of unbound Mff on the OMM. *T* and *M* bind reversibly with rate constants *k*_1_ and *k*_−1_ to form Drp1-Mff complexes. *C*_1_—concentration of these Drp1-Mff complexes which are considered an oligomer of size one. These complexes bind sequentially with rate constants *k*_+_ and *k*_−_ to form oligomers of size 2 through *N*. The fission rate *f*(*i*) is size-dependent. After a fission event, *T* and *M* return to their respective unbound pools. Created with BioRender.com.

Note that the association rate *k*_+_ and dissociation rate *k*_−_ do not depend on the size of the oligomer. Hence a *C*_1_ is just as likely to bind to an oligomer of size five as to an oligomer of size twenty and likewise for the unbinding process. The oligomer construction process along with the assumption that *k*_+_ and *k*_−_ are constant are motivated by the Drp1 oligomerization model in [14] and an actin filamentation model seen in [21]. Fig 3 is a reaction schematic of the model including recruitment, binding of Drp1 to Mff, oligomerization, and finally fission.

Within the mechanism, Drp1 travels from the cytosol to the OMM and back to the cytosol, so the Fission Model can be thought of as a compartmental model; one compartment is the cytosol, and the other compartment is the OMM. The two compartments have different effective spaces, so the Fission Model involves a dimensionless spatial correction term *γ*, which is the ratio of the effective space of the OMM to the effective space of the cytosol. Since the cytosol has a larger effective space than the OMM, the recruitment of Drp1 tetramers to the OMM minimally affects the concentration of Drp1 in the cytosol. Thus the cytosolic concentration of Drp1 (*T*) changes much more slowly over time compared to concentrations on the OMM.

Using the law of mass action, we can build a system of nonlinear ODEs based off Fig 3. There are three terms in Eq (2) that track the fluctuations in the concentration of cytosolic Drp1 tetramers. The concentration of cytosolic Drp1 decreases when Drp1 binds to Mff, and the concentration increases after a fission event or if a *C*_1_ breaks apart (Fig 3). The decrease in concentration is accounted for with the −*k*_1_*TM* term in Eq (2). The increase after a fission event is accounted for with the summation $\sum_{i=1}^{N}\;if\left(i\right)C_{i}$ where *N* is the maximum oligomer size. In the summation, *i* refers to the size of an oligomer which corresponds to the number of Drp1 tetramers that make up that oligomer. Lastly, the *k*_−1_*C*_1_ term accounts for the increase from a *C*_1_ breaking into *T* and *M*. The spatial correction term *γ* accounts for the difference in the effective space of the cytosol and the OMM. Eq (3) behaves very similarly to Eq (2) except their is no *γ* term since we have already accounted for the difference in effective spaces in the previous equation.

Eq (4) tracks the concentration of Drp1-Mff complexes (*C*_1_) over time. These complexes function as the building blocks for larger oligomers. If a Drp1 tetramer binds with an Mff protein, then *C*_1_ increases. If a Drp1 tetramer detaches from an Mff protein then *C*_1_ decreases. These two scenarios are accounted for with the terms *k*_1_*TM* and −*k*_−1_*C*_1_ in Eq (4). If an oligomer of size two breaks apart, then it breaks into two oligomers of size one. This scenario is accounted for with the term 2*k*_−_*C*_2_. Recall an oligomer is built one Drp1-Mff complex at a time (Eq (1)). Therefore, any oligomer larger than size two can only lose a single Drp1-Mff complex in a single reaction. The term $k_{-}\sum_{i=3}^{N}C_{i}$ accounts for the increase in *C*_1_ that would occur if any oligomer larger than size two loses a single Drp1-Mff complex. Similarly, the system loses two Drp1-Mff complexes if they bind to make an oligomer of size two. When building any size larger than two, only a single Drp1-Mff complex is lost in each reaction. These scenarios are accounted for with the term $-k_{+}\left(2C_{1}^{2}+\sum_{i=2}^{N-1}\;C_{i}C_{1}\right)$.

Eq (5) represents the ODE for the concentration of an oligomer of size *i* where 2 ≤ *i* < *N*. These equations track the changes in the concentrations of oligomers as they grow on the OMM. An oligomer of size *i* is built by either an oligomer of size *i*−1 binding with a Drp1-Mff complex or by an oligomer of size *i* + 1 losing a Drp1-Mff complex. These reactions are accounted for with the terms *k*_+_*C*_*i*−1_*C*_1_ and *k*_−_*C*_*i*+ 1_ in Eq (5). Similarly an oligomer of size *i* can be used to build an oligomer of size *i* + 1 if it binds with a Drp1-Mff complex, or a complex could detach creating an oligomer of size *i* − 1. Both of these scenarios would decrease the concentration of oligomers of size *i*, so they are accounted for with the terms −*k*_+_*C*_*i*_*C*_1_ and *k*_−_*C*_*i*_ in Eq (5). The final term in this equation is the fission term which accounts for the loss of an oligomer due to a fission event. The parameter *f*(*i*) is the fission rate which is a function of oligomer size.

Lastly, Eq (6) tracks the concentration of oligomers of size *N* which is the maximum oligomer size in this system. The concentration of oligomers of size *N* increases if an oligomer of size *N* − 1 binds with a Drp1-Mff complex (*k*_+_*C*_*N*−1_*C*_1_) and decreases if a Drp1-Mff complex detaches from an oligomer of size *N* (*k*_−_*C*_*N*_). The concentration will also decrease if an oligomer of size *N* becomes an active fission complex and causes a fission event (*f*(*N*)*C*_*N*_).

$$\begin{array}{c} \dot{T}=\gamma (k_{-1}C_{1}-k_{1}TM+\sum_{i=1}^{N}if\left(i\right)C_{i}), \end{array}$$ (2)

$$\begin{array}{c} \dot{M}=k_{-1}C_{1}-k_{1}TM+\sum_{i=1}^{N}if\left(i\right)C_{i}, \end{array}$$ (3)

$$\begin{array}{c} {\dot{C}}_{1}=k_{1}TM-k_{-1}C_{1}+k_{-}(2C_{2}+\sum_{i=3}^{N}C_{i})-k_{+}(2C_{1}^{2}+\sum_{i=2}^{N-1}C_{i}C_{1}), \end{array}$$ (4)

$$\begin{array}{c} {\dot{C}}_{i}=k_{+}C_{i-1}C_{1}+k_{-}C_{i+1}-k_{-}C_{i}-k_{+}C_{i}C_{1}-f\left(i\right)C_{i}\;\;\;2\leq i<N, \end{array}$$ (5)

$$\begin{array}{c} {\dot{C}}_{N}=k_{+}C_{N-1}C_{1}-k_{-}C_{N}-f\left(N\right)C_{N}. \end{array}$$ (6)

The Fission Model has the following conserved quantities:

$$\begin{array}{c} T=T+\gamma (\sum_{i=1}^{N}iC_{i}), \end{array}$$ (7)

$$\begin{array}{c} M=M+\sum_{i=1}^{N}iC_{i} \end{array}$$ (8)

where T is the total concentration of tetrameric Drp1, M is the total concentration of Mff on the OMM.

The corkscrew constriction process of Drp1 oligomers [22, 23] requires full encircling of the mitochondrion. This suggests the fission rate function *f*(*i*) will be zero below some threshold length. Thus the fission rate function is defined as a piece-wise constant function where the fission rate below some threshold *ℓ* is zero and the fission rate above *ℓ* is a constant value *a* with units 1/*s*.

$$\begin{array}{c} f(i)=\{\begin{array}{c} 0,\;1\leq i\leq \ell ,\\ a,\;\ell <i<N. \end{array} \end{array}$$ (9)

Michalska et. al. in [14] used the TrackMate ImageJ plugin to detect Drp1-GFP spots on mitochondria. They used the intensity of these spots to determine the number of Drp1 molecules and found “the average number of Drp1 molecules forming a functional fission complex to be approximately 100” [14]. Therefore the Fission Model sets *ℓ* = 25 in the numerical solutions discussed in this paper. This restricts fission events to be initiated by oligomers of size 26 and greater. In the simulations below, the fission rate for these larger oligomers is set to $a=5\frac{1}{s}$. This relatively large value ensures the rate of fission does not act as a rate limiting step within the model allowing us to focus on the dynamics of oligomerization. An exploratory parameter sensitivity analysis will be performed later to determine how different choices of *a* within the same magnitude affect the total fission rate. Lastly, we let *N* = 30 because the numerical solutions show the concentration of oligomers larger than size 26 is very small. We would like to note that changes in *ℓ* and *N* do not alter the qualitative behavior of the system as long as N is sufficiently large. Also, if *a* is chosen to be orders of magnitude smaller, the system will build larger oligomers but causes fission events to occur on an unrealistically slow timeline. A smaller value for *a* also obscures the oligomerization process because the simulations show very few fission events. Hence, the system loses the cyclic, replenishing effect of Mff returning to its unbound pool.

With these choices for *f*(*i*), *N*, and *ℓ*, the system of ODEs becomes

$$\begin{array}{c} \dot{T}=\gamma (k_{-1}C_{1}-k_{1}TM+a\sum_{i=26}^{30}iC_{i}), \end{array}$$ (10)

$$\begin{array}{c} \dot{M}=k_{-1}C_{1}-k_{1}TM+a\sum_{i=26}^{30}iC_{i}, \end{array}$$ (11)

$$\begin{array}{c} {\dot{C}}_{1}=k_{1}TM-k_{-1}C_{1}+k_{-}(2C_{2}+\sum_{i=3}^{30}C_{i})-k_{+}(2C_{1}^{2}+\sum_{i=2}^{29}C_{i}C_{1}), \end{array}$$ (12)

$$\begin{array}{c} {\dot{C}}_{i}=\{\begin{array}{c} -k_{+}C_{1}\left(C_{i}-C_{i-1}\right)+k_{-}\left(C_{i+1}-C_{i}\right)\;2\leq i\leq 25\\ -k_{+}C_{1}\left(C_{i}-C_{i-1}\right)+k_{-}\left(C_{i+1}-C_{i}\right)-aC_{i}\;25<i\leq 29, \end{array} \end{array}$$ (13)

$$\begin{array}{c} {\dot{C}}_{30}=k_{+}C_{29}C_{1}-k_{-}C_{30}-aC_{30}. \end{array}$$ (14)

The conserved quantities in Eqs (7) and (8) remain the same except the upper bound on the summations is now set to 30.

## 3 Results

### 3.1 Numerical solutions of the Fission Model

The lack of information in the literature about the association and dissociation rate constants between Drp1 and Mff (*k*_1_, *k*_−1_), oligomerization on the OMM (*k*_+_, *k*_−_), and the rate of mitochondrial fission (*f*(*i*)) motivated our investigation of how different parameter choices affect the total fission rate with the goal of identifying regulatory parameters which fine tune mitochondrial fission. The total fission rate (TFR) is the total number of fission events per second, and it has units *nM*/*s*. Thus the TFR as a function of time is

$$\begin{array}{c} TFR\left(t\right)=a\sum_{i=26}^{30}C_{i}\left(t\right) \end{array}$$ (15)

where *a* is the fission rate from Eq (9) with units 1/*s*.

**Fig 4:**
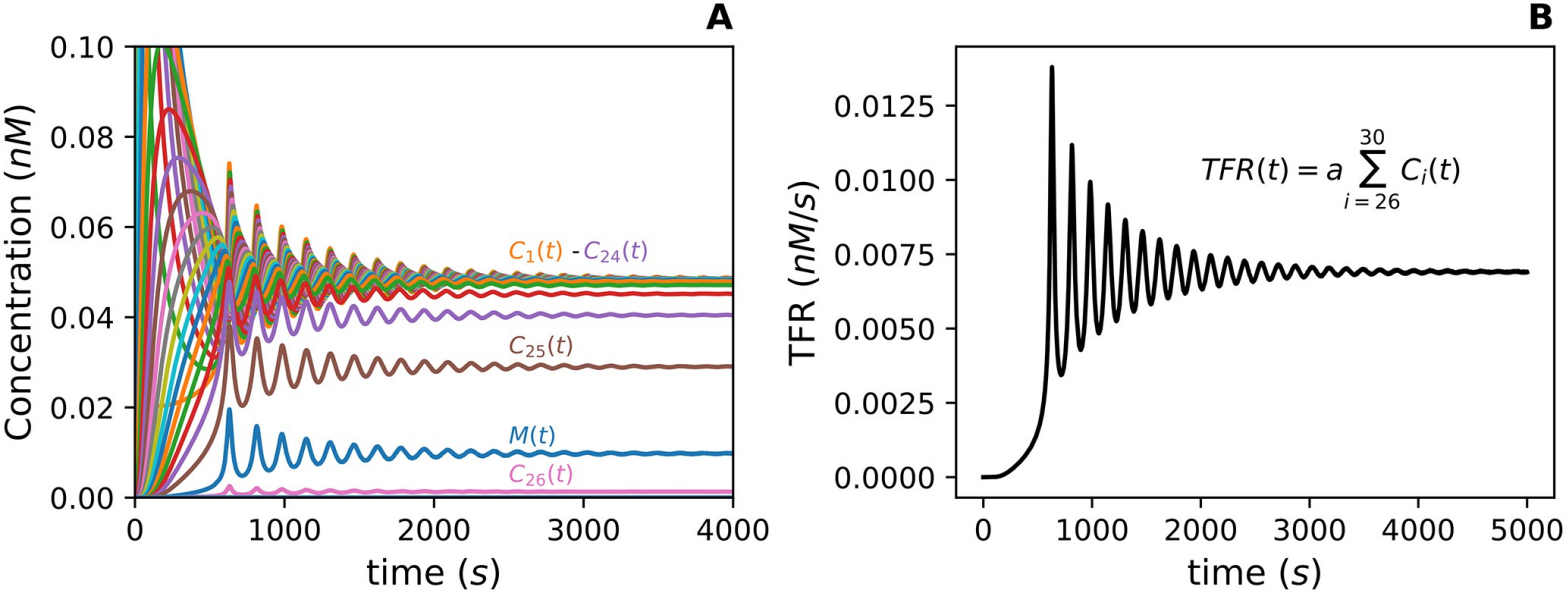
**(A)** Numerical Solutions for *M*(*t*) and *C*_*i*_(*t*) for 1 ≤ *i* ≤ 30. The solution curves for 27 ≤ *i* ≤ 30 are very close to zero. The solution for *T*(*t*) is not shown since its concentration varies minimally over the time course. There is a delay in the initial spike of the larger oligomer sizes, e.g. *C*_25_(*t*). This is due to the restriction that oligomers are assembled one Drp1-Mff complex at a time. **(B)** The total fission rate (TFR) as a function of time is the sum of the fission rates for all oligomers large enough to form an active fission complex.

The initial conditions for the numerical solutions assume the total concentration of Drp1 tetramers (T) begins in the unbound cytosolic pool, and the total concentration of Mff (M) is in the unbound pool on the OMM which implies the initial concentration of oligomers of any size, *C*_*i*_ for 1 ≤ *i* ≤ 30, is 0. The parameter values for the numerical solutions and the TFR curve shown in Fig 4 are listed in Table 1. The values in Table 1 were chosen so that the qualitative behavior of the numerical solutions in Fig 4A allowed for an investigation of the oligomerization process. The model focuses on the oligomerization process in order to better understand how changes upstream of a fission event affect the number of fission events per second.

**Table 1:** Parameter Choices for the Fission Model.

*k*_1_ = 1 (*nMs*)^−1^	*k*_−1_ = 0.02 *s*^−1^
*k*_+_ = 5 (*nMs*)^−1^	*k*_−_ = 0.1 *s*^−1^
*a* = 5 *s*^−1^	*γ* = 0.1
T=20nM	M=15nM

The numerical solutions exhibit damped oscillations that appear to reach a steady-state (Fig 4A). These oscillations are a consequence of the cyclic nature of the system caused by Mff returning to an unbound state after a fission event. Notice the numerical solution curves for various oligomer sizes oscillate in sync with the solution curve for Mff. This is because the availability of Mff to bind with Drp1 dictates the concentration of *C*_1_ and by extension, the systems oscillations. The numerical solutions for larger oligomer sizes, e.g. *C*_25_(*t*), experience a delay in their initial spike (Fig 4A) since the Fission Model builds oligomers one Drp1-Mff complex at a time. In other words, an oligomer of size 24 needs to exist before an oligomer of size 25 can be built. The solution curves for *C*_*i*_(*t*) for 26 < *i* ≤ 30 are not visible in Fig 4A because their concentration levels are very close to zero. There is also an initial decrease in the concentration of Mff before any other oscillations occur which is not visible in Fig 4A. This is due to the initial condition that the concentration of every oligomer size at time zero is zero. The numerical solutions in Fig 4A do not contain the solution for *T*(*t*) because over the time course, the solution *T*(*t*) varies very little from its initial concentration. Fig 4B shows the TFR as a function of time computed from the numerical solutions *C*_*i*_(*t*) for 26 ≤ *i* ≤ 30 along with the fission rate, *a*, from Table 1. The TFR curve mimics the damped oscillatory behavior of the numerical solutions.

### 3.2 An exploratory parameter sensitivity analysis

**Fig 5:**
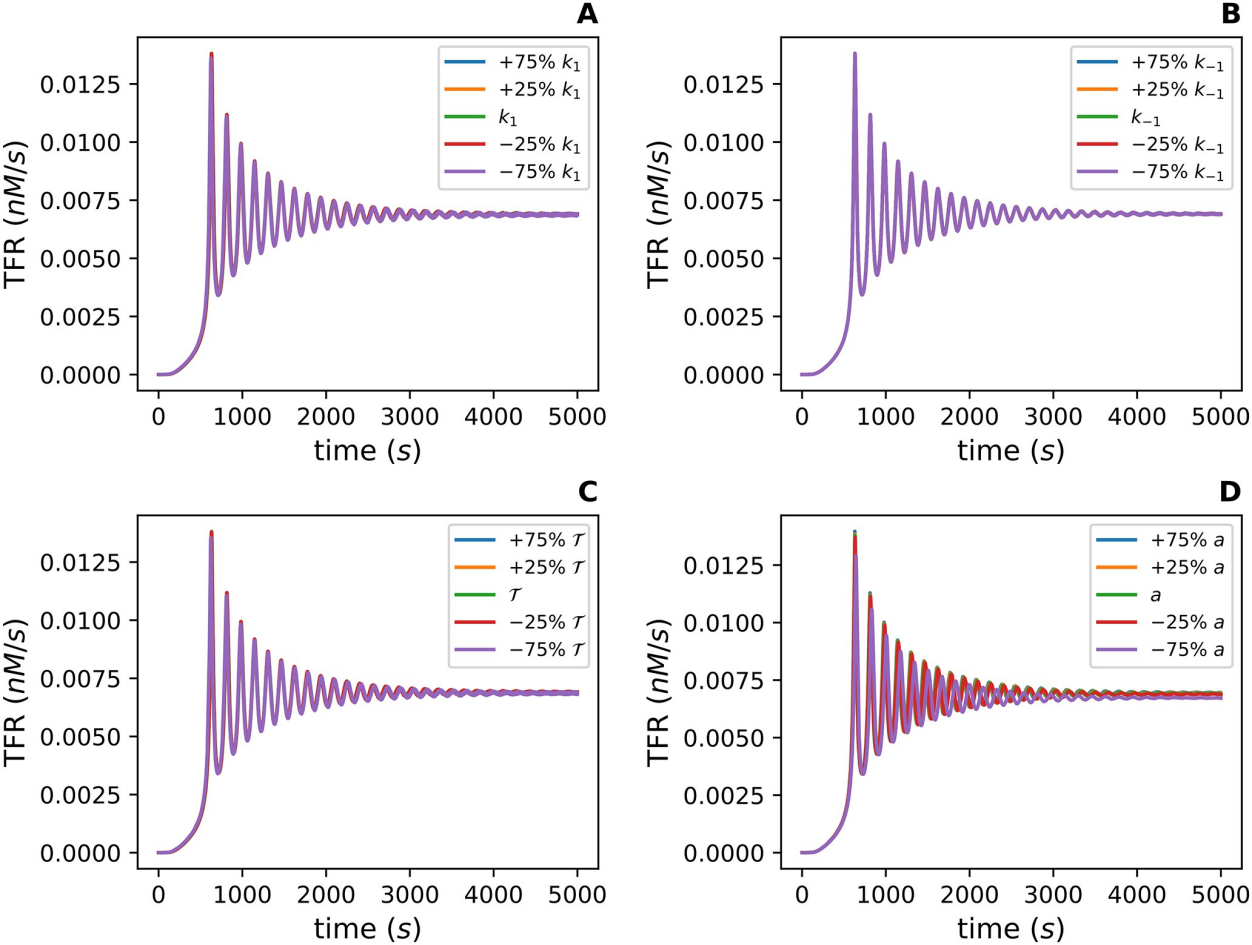
An exploratory parameter sensitivity analysis for *k*_1_, *k*_−1_, T, and *a* with regard to the total fission rate (TFR). The TFR is defined as a $a\sum_{i=26}^{30}C_{i}\left(t\right)$. **(A), (B), (C)** and **(D)** depict how the TFR changes with ±25% and ±75% of the value of *k*_1_, *k*_−1_, T, and *a* from Table 1 respectively.

An exploratory parameter sensitivity analysis was conducted to determine how different parameter values affect the qualitative behavior of the TFR curve. The parameter values in Table 1 were varied by ±25% and ±75%. The parameters were changed one at a time, and a TFR curve was computed for each new parameter value. We used large parameter varations for this investigation since our parameter values were chosen with minimal prior knowledge about their true values. This exploratory investigation showed *k*_1_ (Fig 5A), *k*_−1_ (Fig 5B), and T (Fig 5C) minimally affect the TFR. In fact, the TFR curve for each value of *k*_1_, *k*_−1_, T is nearly identical to the original TFR curve. This is illustrated in Fig 5A, 5B and 5C by all the curves sitting on top of one another.

As expected, the total concentration of Drp1 (T) has a minimal effect on the TFR because the amount of Drp1 tetramers in the cytosol is larger than the amount of Mff on the OMM due to the cytosol having a larger effective space. Therefore, an increase or decrease in the total concentration of Drp1 has minimal effect on Drp1-Mff interactions and consequently minimal effect on the TFR.

With ±25% and ±75% of the fission rate *a* (Fig 5D), the period and amplitude of the TFR vary slightly, but the qualitative behavior is unaffected. In summary, *k*_1_, *k*_−1_, T, and *a* do not appear to affect the qualitative behavior of the TFR in this initial analysis.

**Fig 6:**
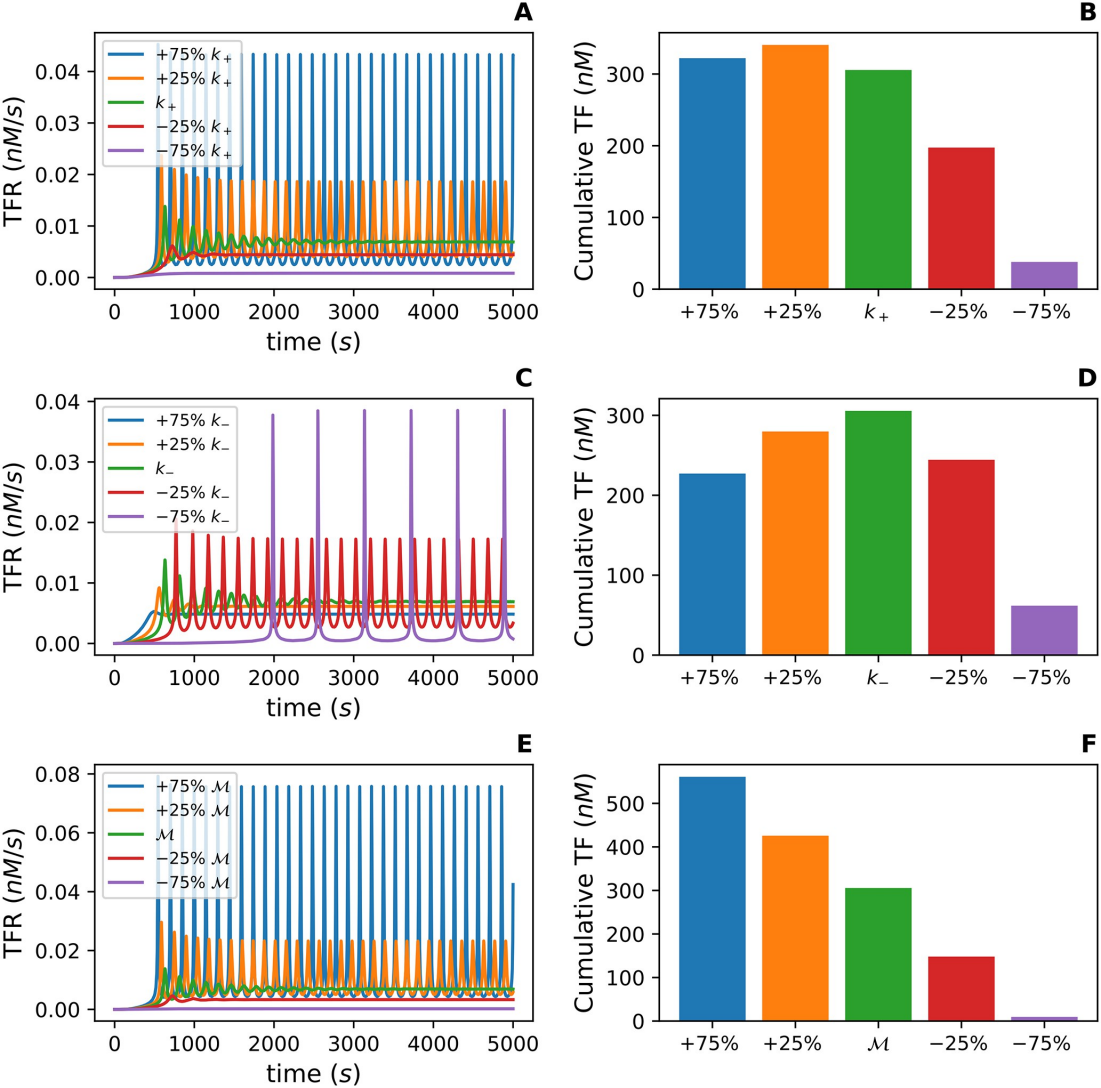
An exploratory parameter sensitivity analysis for *k*_+_, *k*_−_, and M with respect to the total fission rate (TFR). The TFR is defined as $a\sum_{i=26}^{30}C_{i}\left(t\right)$. **(A), (C),** and **(E)** depict how the TFR varies with ±25% and ±75% of the value of *k*_+_, *k*_−_, and M in Table 1 respectively. **(B), (D),** and **(F)** shows the cumulative TF associated with each parameter variation. The cumulative TF is computed by integrating the TFR function from 0 to 5000 seconds for each parameter variation.

In contrast, an increase in *k*_+_ by 25% and 75%, transitions the TFR from damped oscillations to sustained oscillations (Fig 6A). A decrease in *k*_+_ by 25% decreases the amplitude of the damped oscillations and decreases the time necessary to reach the steady-state TFR value (Fig 6A). Recall that *k*_+_ is the association rate constant for Drp1-Mff complexes and larger oligomers on the OMM (Eq (1)). Thus *k*_+_ can be conceptualized as the Fission Model’s desire to build large oligomers. If *k*_+_ is too small, then the system will not build oligomers large enough to form an active fission complex. This is illustrated by the curve associated with a 75% decrease in *k*_+_ (Fig 6A). Fig 6B shows the cumulative total fission (TF) for each variation of *k*_+_. These values are the result of integrating the TFR function from time 0 to 5000 for each simulation. Interestingly, an increase in *k*_+_ does not always yield a higher cumulative TF. While the + 75% curve (Fig 6B) has a higher amplitude, the frequency and width of the peaks has decreased resulting in a lower cumulative TF.

Recall *k*_−_ is the dissociation rate of Drp1-Mff complexes from a larger oligomer (Eq (1)). An increase in *k*_−_ by 25% and 75% maintains the damped oscillations and decreases the necessary time to reach the steady-state TFR value (Fig 6C). In contrast, when *k*_−_ is decreased by 25% the qualitative behavior of the TFR curve transitions to sustained oscillations (Fig 6C). With a 75% decrease, the period drastically increases compared to a 25% decrease (Fig 6C). The cumulative TF also plummits (Fig 6D). One might expect if we continued to decrease *k*_−_, then the cumulative TF would increase and behave similarly to what was seen with increases in *k*_+_. Recall that *k*_−_ is conceptualized as the desire of a single complex *C*_1_ to detach from an oligomer. Therefore, if *k*_−_ is too small, the pool of available *C*_1_ becomes depleted, and the model creates medium sized oligomers that are not large enough to form an active fission complex. This drastically delays fission events illustrated by the curve associated with a 75% decrease in *k*_−_ (Fig 6C). Therefore, if *k*_−_ becomes too small, then the number of fission events over time will decrease drastically.

In Fig 6E, the ±25% and ±75% variations in the total concentration of Mff (M) yield similar results to those seen in Fig 6A. One difference, however, is that the cumulative TF monotonically increases as M increases (Fig 6F). This is because, within the model, Drp1 is in excess in the cytsol, so an increase in the total concentration of Mff allows for more Drp1 to bind to the OMM. This behavior is consistent with the experimental results that over expression of Mff induces mitochondrial fragmentation with increased Drp1 recruitment to the mitochondria [13, 20].

Through this initial investigation, *k*_1_, *k*_−1_, *a*, and T appear to have minimal effect on the system’s TFR while variations in *k*_+_, *k*_−_, and M appear to drastically affect the qualitative behavior of TFR. We will further quantify these effects in terms of the cumulative TF with a variance-based global sensitivity analysis.

### 3.3 Variance-based global sensitivity analysis with Sobol’s method

A variance-based global sensitivity analysis (GSA) determines each parameter’s contribution to the variation in the model output. For this variance-based GSA, we use Sobol’s method and compute the first-order, total-order, and second-order sensitivity indices. The model output is the cumulative TF. The following summary for a variance-based GSA with Sobol’s method is motivated by the variance-based GSA section from [24]. Suppose *Y* = *f*(*X*_1_, *X*_2_, …, *X*_*k*_) where *Y* is the model output, *X*_1_, *X*_2_,…,*X*_*k*_ are the model parameters. The first-order sensitivity index *S*_*i*_ for parameter *X*_*i*_ is defined as

$$\begin{array}{c} S_{i}\equiv \frac{V(E\left(Y|X_{i}=x_{i}^{*}\right))}{V(Y)}, \end{array}$$ (16)

where E is the expected value and V is the variance. The numerator is approximated by first computing the expected value of the model output for all possible parameter values while maintaining *X*_*i*_ at a fixed value $x_{i}^{*}$. This process is repeated for all simulated parameter values of $x_{i}^{*}$. Then, the variance of all expected values is computed and divided by the variance of the model output. The first-order index is a parameter’s individual contribution to the overall variation in the model output. Higher order interactions between parameters come into play with the total-order sensitivity index. The total-order sensitivity index for a parameter *X*_*i*_ is defined as

$$\begin{array}{c} S_{i}^{T}\equiv 1-\frac{V(E\left(Y|X_{\;i}=x_{\;i}^{*}\right))}{V(Y)}, \end{array}$$ (17)

The quotient in Eq (17) represents the variation coming from all parameters excluding *X*_*i*_. Thus subtracting this quantity from 1 gives the total contribution of *X*_*i*_. In other words, the total-order sensitivity index represents the variation coming from *X*_*i*_ individually along with the variation coming from higher order interactions between *X*_*i*_ and other parameters. If the total-order sensitivity index is much larger than the first-order sensitivity index, then higher order interactions are likely contributing to the variation in the model output. The second-order sensitivity index measures the contribution from second-order interactions and is computed for each pair of parameters.

For this GSA, the first, second, and total-order sensitivity indices were computed numerically using the Python package *SALib*, specifically the functions from the modules SALib.sample.sobol and SALib.analyze.sobol [25, 26]. The first module generates a pseudo-random set of *N* ⋅ (2*D* + 2) number of values for each parameter where *N* is 2^x^ for some integer *x* and *D* is the number of parameters in question. The values which makeup these sets are sampled from distributions assigned by the user. For this analysis, *x* equals 11 and *D* is either 4 (*Case 1*) or 5 (*Case 2*). This gives us two sample sizes, 20, 480 4-tuple samples for *Case 1* and 24, 576 5-tuple samples for *Case 2*. The second package uses these samples to compute the first- and total-order sensitivity indices according to the methods described by [27]. The second-order sensitivity indices are computed according to the method described by [28].

The null hypothesis for a variance-based GSA is that a parameter does not contribute to the variation in the model output. This translates to a Sobol index of zero. Therefore, if the 95% confidence interval for an index contains zero, then we fail to reject the null hypothesis. The results for the Fission Model are broken up into two cases:

*Case 1*: The parameters investigated in this case are *k*_1_, *k*_−1_, *k*_+_, and *k*_−_. The distribution assigned to the first three parameters is the uniform distribution from (0, 10). The distribution assigned to *k*_−_ is the uniform distribution from (0, 1). Originally, *k*_−_ was also assigned the uniform distribution from (0, 10), but the GSA results showed the majority of the variation in the cumulative TF occurs when *k*_−_ is between 0 and 1, and there is minimal variation for larger values of *k*_−_. Thus, we repeated the analysis and assigned *k*_−_ the uniform distribution from (0,1). Uniform distributions were chosen since we have no prior knowledge about the true values of these parameters.

*Case 2*: The parameters investigated in this case are *k*_1_, *k*_−1_, *k*_+_, *k*_−_, and M. The distribution assigned to *k*_1_, *k*_−1_, and *k*_+_ is the uniform distribution from (0,10). The distribution assigned to M is the uniform distribution from (0,30) since M=15 in the numerical solutions (Table 1). Lastly, the distribution assigned to *k*_−_ is the uniform distribution from (0, 1). This is again because we observed the largest cumulative TF values occur for values of *k*_−_ between 0 and 1.

The first-order indices for *Case 1* (Table 2) show the majority of the model output’s variation comes from *k*_+_ and *k*_−_. These parameters also have the largest increase between their first- and total-order indices. This increase is due to their second-order interactions with each other *k*_+_ : *k*_−_ (Table 3). The second-order interaction between *k*_+_ and *k*_−_ is the only second-order interaction we can reject the null hypothesis for in *Case 1*. With the inclusion of M in *Case 2*, the first-order indices for *k*_+_ and *k*_−_ decrease compared to *Case 1*, and we now fail to reject the null hypothesis for the second order interaction of *k*_+_ : *k*_−_ (Tables 2 and 3). In *Case 2*, the parameter M is the largest contributor to the variation in the cumulative TF, and the significant second-order interactions are now M with *k*_+_ and M with *k*_−_ (Tables 2 and 3).

**Table 2:** The first- and total-order sensitivity indices for *Case 1* were calculated from 20, 480 4-tuple samples and include a 95% confidence interval. The indices for *Case 2* were calculated from 24, 576 5-tuple samples. First-order indices characterize a parameters individual contribution to the variation in the model output. Total-order indices characterize a parameters total contribution to the variation in the model output which includes higher order interactions.

*Case 1:*	*S*_1_	*S*_*T*_
*k*_1_	0.0004 ± 0.0021	0.0012 ± 0.0008
*k*_−1_	0.0000 ± 0.0004	0.0002 ± 0.0002
*k*_+_	0.7653 ± 0.0580	0.8742 ± 0.0623
*k*_−_	0.1035 ± 0.0267	0.2317 ± 0.0280
*Case 2:*	*S*_1_	*S*_*T*_
*k*_1_	−0.0009 ± 0.0020	0.0012 ± 0.0014
*k*_−1_	−0.0001 ± 0.0003	0.0000 ± 0.0000
*k*_+_	0.2116 ± 0.0444	0.4887 ± 0.0523
*k*_−_	0.0240 ± 0.0183	0.1834 ± 0.0371
M	0.4452 ± 0.0630	0.7181 ± 0.0756

**Table 3:** The second-order Sobol indices for *Case 1* were calculated from 20, 480 4-tuple samples and include a 95% confidence interval. The indices for *Case 2* were calculated from 24, 576 5-tuple samples. Second-order indices quantify the variation in the model output which comes from second-order interactions between parameters.

*Case 1:*	*S*_2_		*S*_2_
*k*_1_ : *k*_−1_	−0.0006 ± 0.0020	*k*_1_ : *k*_+_	−0.0005 ± 0.0022
*k*_1_ : *k*_−_	0.0005 ± 0.0025	*k*_−1_ : *k*_+_	−0.0000 ± 0.0007
*k*_−1_ : *k*_−_	0.0009 ± 0.0011	*k*_+_ : *k*_−_	0.1189 ± 0.0727
*Case 2:*	*S*_2_		*S*_2_
*k*_1_ : *k*_−1_	0.0013 ± 0.0037	*k*_1_ : *k*_+_	0.0014 ± 0.0033
*k*_1_ : *k*_−_	0.0020 ± 0.0051	$k_{1}:M$	0.0008 ± 0.0034
*k*_−1_ : *k*_+_	0.0003 ± 0.0004	*k*_−1_ : *k*_−_	0.0003 ± 0.0004
$k_{-1}:M$	0.0000 ± 0.0004	*k*_+_ : *k*_−_	0.0110 ± 0.0627
$k_{+}:M$	0.1684 ± 0.0868	$k_{-}:M$	0.0696 ± 0.0433

**Fig 7:**
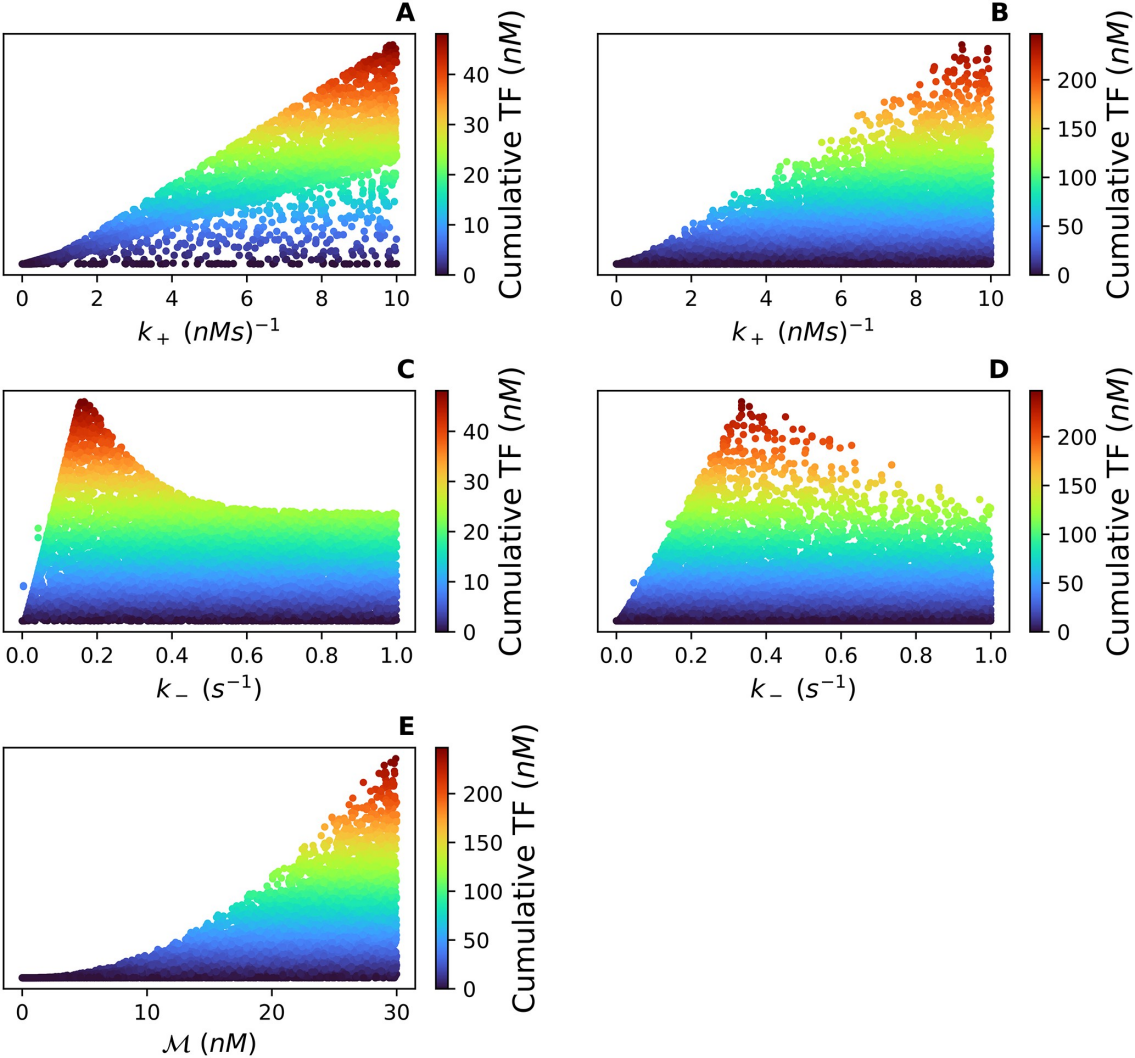
**(A)** and **(B)** show *k*_+_ vs the cumulative TF for *Case 1* and *Case 2* respectively. **(C)** and **(D)** show *k*_−_ vs the cumulative TF for *Case 1* and *Case 2* respectively. **(E)** shows M vs the cumulative TF for *Case 2*. Note that each point represents a 4-tuple (or 5-tuple) of parameter values for (*k*_1_, *k*_−1_, *k*_+_, *k*_−_) (or $\left(k_{1},k_{-1},k_{+},k_{-},M\right)$) and the corresponding cumulative TF specific to that 4-tuple (or 5-tuple). The range of the cumulative TF is case specific with a larger range of values for *Case 2*. This increase in the cumulative TF is because *Case 2* allows for variations in the total concentration of Mff (M).

For each simulation in *Case 1*, a 4-tuple of parameter values is selected (a 5-tuple in *Case 2*) and the cumulative TF is computed. In Fig 7, we show scatter plots of the observed cumulative TF values vs each of the significant parameters’ values. The reader should note that all 4 (or 5) of the parameter values are being varied simultaneously. Also, the range of the cumulative TF is case specific. The cumulative TF has a much larger range in *Case 2* since this case allows for variations in the total concentration of Mff (M).

Across both cases, the larger values of *k*_+_ are associated with a larger cumulative TF (Fig 7A and 7B). In contrast, *k*_−_ has a more nuanced range of values associated with the largest cumulative TF (Fig 7C and 7D). The range of cumulative TF values is much larger in *Case 2* causing the tuples to appear more scattered than in *Case 1*. Recall from the exploratory parameter sensitivity analysis, the cumulative TF monotonically increases as M increases (Fig 6F). Thus the increase in the range of cumulative TF is likely due to the increased range of M.

**Fig 8:**
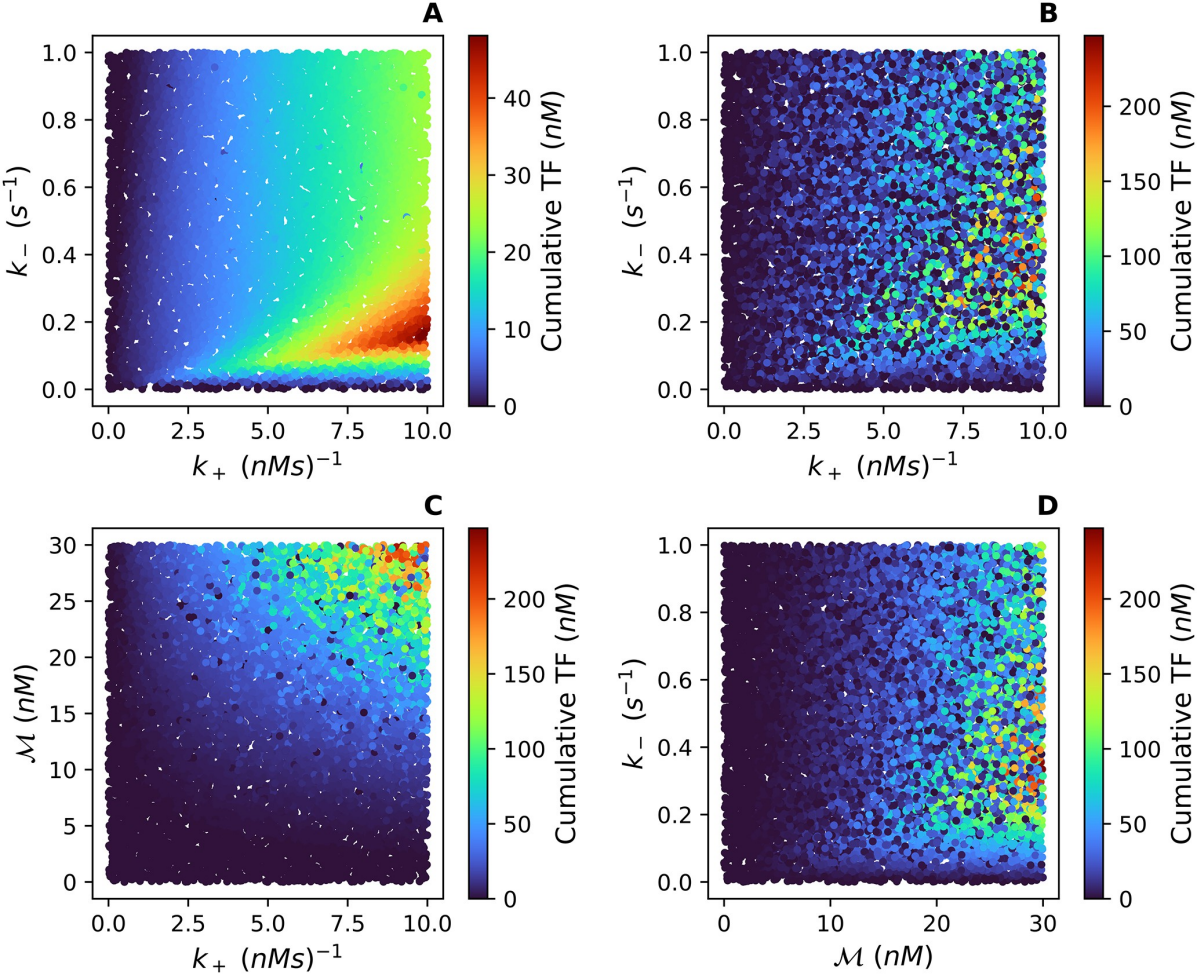
**(A)** shows *k*_+_ vs *k*_−_ and the corresponding cumulative TF for the 4-tuples from *Case 1*. **(B)** shows *k*_+_ vs *k*_−_ and the corresponding cumulative TF for the 5-tuples from *Case 2*. **(C)** shows *k*_+_ vs M and the corresponding cumulative TF associated with each 5-tuple from *Case 2*. **(D)** shows *k*_−_ vs M and the corresponding cumulative TF associated with each 5-tuple from *Case 2*. The range of the cumulative TF is case specific with a larger range of values for *Case 2*. This increase in the cumulative TF is because *Case 2* allows for variations in the total concentration of Mff (M).

The scatter plots in Fig 8 illustrate the significant second-order interactions in both cases. In *Case 1*, the second-order interaction between *k*_+_ and *k*_−_ is significant and Fig 8A shows the range of values for *k*_+_ and *k*_−_ which produce the largest cumulative TF. In *Case 2*, however, the confidence interval for the second-order index of *k*_+_ : *k*_−_ contains zero, so we fail to reject the null hypothesis. Fig 8B illustrates how the addition of M increases the variability of the cumulative TF enough to begin to obscure the second-order interaction of *k*_+_ : *k*_−_, and the interactions of $k_{+}:M$ and $k_{-}:M$ become the main second-order contributors to the variation. The primarily radial/angular structure seen in Fig 8A, 8B and 8D suggests that the ratios *k*_+_/*k*_−_ and $M/k_{-}$ are relevant. Similarly, the primarily hyperbolic structure in Fig 8C suggests that the product $k_{+}M$ is also meaningful. Taken together, these results lead us to hypothesize that a dimensionless parameter $\mu =\frac{k_{+}M}{k_{-}}$ is the strongest determinant of cumulative TF.

In summary, the total concentration of Mff on the OMM (M) along with the association rate (*k*_+_) and dissociation rate (*k*_−_) between oligomers on the OMM contribute more variation to the cumulative TF than the association rate (*k*_1_) and dissociation rate (*k*_−1_) between Drp1 with Mff. This is consistent with the results from the exploratory parameter sensitivity analysis which showed *k*_+_, *k*_−_, and M affect the qualitative behavior of the TFR curve (Fig 6). Thus a combination of these three parameters, specifically the ratio $\mu =\frac{k_{+}M}{k_{-}}$ could serve as a regulatory parameter for the Fission Model. Next, through a nondimensionalization, we determine the effect of *μ* on the qualitative behavior of the TFR and on the variation in the cumulative TF.

### 3.4 A nondimensionalization of the Fission Model

In this section, we nondimensionalize the Fission Model in order to investigate a dimensionless parameter *μ* which depends on *k*_+_, *k*_−_, and M. The new dimensionless variables for the nondimensionalization of the Fission Model are

$$\begin{array}{c} z=\frac{T}{M},\;\text{dimensionless}\;\text{cytosolic}\;\text{Drp1},\\ y=\frac{M}{M},\;\text{dimensionless}\;\text{unbound}\;\text{Mff},\\ x_{i}=\frac{C_{i}}{M},\;1\leq i\leq 30,\;\text{dimensionless}\;\text{oligomers},\\ \eta =tk_{-},\;\text{dimensionless}\;\text{time}. \end{array}$$

The dimensionless system is derived in two steps: the first step applies a derivative with respect to time *t* to the new dimensionless variables, and the second step applies a derivative with respect to dimensionless time *η*.

Step 1:

$$\begin{array}{c} \frac{dz}{dt}=\frac{dz}{dT}\frac{dT}{dt}\;=\;\gamma (k_{-1}x_{1}-k_{1}Mzy+a\sum_{i=1}^{30}ix_{i}),\\ \frac{dy}{dt}=\frac{dy}{dM}\frac{dM}{dt}\;=\;k_{-1}x_{1}-k_{1}Mzy+a\sum_{i=1}^{30}ix_{i},\\ \frac{dx_{1}}{dt}=\frac{dx_{1}}{dC_{1}}\frac{dC_{1}}{dt}\;=\;k_{1}Mzy-k_{-1}x_{1}+k_{-}(2x_{2}+\sum_{i=3}^{30}x_{i})-k_{+}M(2x_{1}^{2}+\sum_{i=2}^{29}x_{i}x_{1}),\\ \frac{dx_{i}}{dt}=\frac{dx_{i}}{dC_{i}}\frac{dC_{i}}{dt}\;=\;\{\begin{array}{c} -k_{+}Mx_{1}\left(x_{i}-x_{i-1}\right)+k_{-}\left(x_{i+1}-x_{i}\right),\;2\leq i\leq 25,\\ -k_{+}Mx_{1}\left(x_{i}-x_{i-1}\right)+k_{-}\left(x_{i+1}-x_{i}\right)+ax_{i},\;25<i\leq 29, \end{array}\\ \frac{dx_{30}}{dt}=\frac{dx_{30}}{dC_{30}}\frac{dC_{30}}{dt}\;=\;k_{+}Mx_{29}x_{1}-k_{-}x_{30}-ax_{30}. \end{array}$$

Step 2:

$$\begin{array}{c} \frac{dz}{d\eta }=\frac{dz}{dt}\frac{dt}{d\eta }\;=\;\gamma (\zeta x_{1}-\beta yz+\alpha \sum_{i=26}^{30}ix_{i}), \end{array}$$ (18)

$$\begin{array}{c} \frac{dy}{d\eta }=\frac{dy}{dt}\frac{dt}{d\eta }\;=\;\zeta x_{1}-\beta yz+\alpha \sum_{i=26}^{30}ix_{i} \end{array}$$ (19)

$$\begin{array}{c} \frac{dx_{1}}{d\eta }=\frac{dx_{1}}{dt}\frac{dt}{d\eta }\;=\;\beta yz-\zeta x_{1}+2x_{2}\sum_{i=3}^{30}x_{i}-\mu (2x_{1}^{2}+\sum_{i=2}^{29}x_{i}x_{1}), \end{array}$$ (20)

$$\begin{array}{c} \frac{dx_{i}}{d\eta }=\frac{dx_{i}}{dt}\frac{dt}{d\eta }\;=\;\{\begin{array}{c} -\mu x_{1}\left(x_{i}-x_{i-1}\right)+\left(x_{i+1}-x_{i}\right),\;2\leq i\leq 25,\\ -\mu x_{1}\left(x_{i}-x_{i-1}\right)+\left(x_{i+1}-x_{i}\right)-\alpha x_{i},\;25<i\leq 29, \end{array} \end{array}$$ (21)

$$\begin{array}{c} \frac{dx_{30}}{d\eta }=\frac{dx_{30}}{dt}\frac{dt}{d\eta }\;=\;\mu x_{29}x_{1}-x_{30}\left(1+\alpha \right), \end{array}$$ (22)

where

$$\begin{array}{c} \mu =\frac{k_{+}M}{k_{-}}, \end{array}$$ (23)

$$\begin{array}{c} \alpha =\frac{a}{k_{-}}, \end{array}$$ (24)

$$\begin{array}{c} \beta =\frac{k_{1}M}{k_{-}}, \end{array}$$ (25)

$$\begin{array}{c} \zeta =\frac{k_{-1}}{k_{-}}. \end{array}$$ (26)

The parameter *μ* can be thought of as the maximum build rate over the disassembling rate of oligomers on the outer mitochondrial membrane. Similar to Eqs (7) and (8), the dimensionless conserved quantities are

$$\begin{array}{c} \frac{T}{M}=z+\gamma \sum_{i=1}^{30}ix_{i}, \end{array}$$ (27)

$$\begin{array}{c} 1=y+\sum_{i=1}^{30}ix_{i}, \end{array}$$ (28)

Next, we perform another exploratory parameter sensitivity analysis for the new dimensionless parameters to determine how variations in *μ* affect the qualitative behavior of the TFR.

#### 3.4.1 An exploratory parameter sensitivity analysis for the nondimensionalization

As with the Fission Model, the nondimensionalization has a maximum oligomer size of 30 since on average an active fission complex contains 25 Drp1 tetramers [14]. Using the parameter values in Table 1 and Eqs (23)–(26), the dimensionless parameter values are *μ* = 750, *α* = 50, *β* = 150, and *ζ* = 0.2.

The initial conditions for the nondimensionalization are that the total dimensionless concentration of Drp1 begins in the cytosol, the total dimensionless concentration of Mff begins in an unbound form on the OMM, and *x*_*i*_ = 0 for *i* ≥ 1. Since the nondimensionalization is a rescaling of the original model, the numerical solutions and TFR curve have the same qualitative behavior as seen in Fig 4.

**Fig 9:**
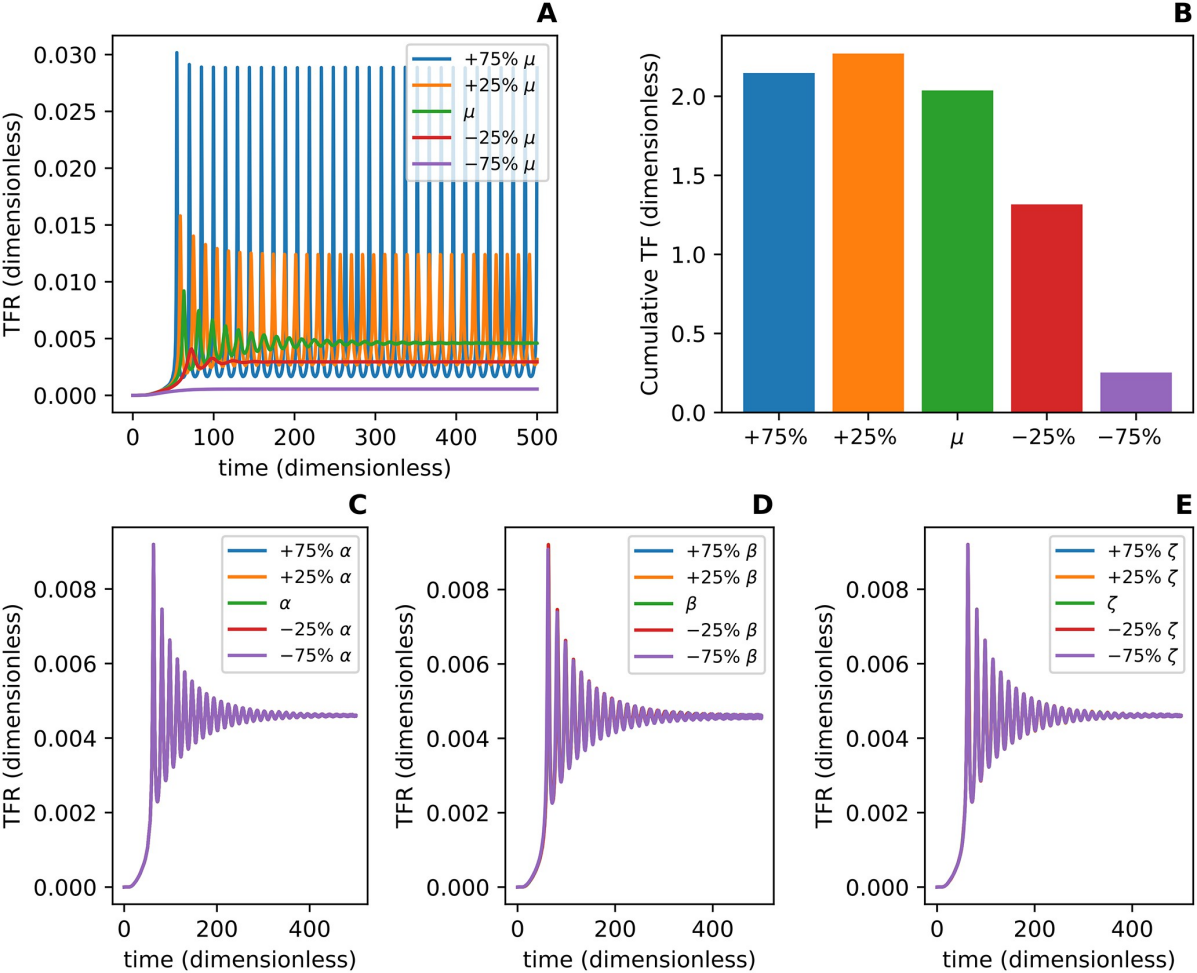
An exploratory parameter sensitivity analysis for the dimensionless parameters *μ*, *α*, *β*, and *ζ*. This analysis is done with respect to the dimensionless total fission rate (TFR) as a function of dimensionless time *η*. The dimensionless TFR is defined as $\alpha \sum_{i=26}^{30}x_{i}\left(\eta \right)$. **(A)** depicts how the dimensionless TFR varies with ±25% and ±75% of the value of *μ*. **(B)** shows the dimensionless cumulative total fission (TF) associated with each value of *μ*. The dimensionless cumulative TF was computed by integrating the dimensionless TFR function from 0 to 500. **(C)**, **(D)**, and **(E)** depict how the dimensionless TFR changes with ±25% and ±75% of the value of *α*, *β*, and *ζ* respectively. The dimensionless TFR is minimally affected by variations in these three parameters which is illustrated by all five curves sitting essentially on top of one another.

To determine whether variations in *μ*, *α*, *β*, or *ζ* affect the behavior of the TFR, we conducted an exploratory parameter sensitivity analysis by varying each parameter one at a time by ±25% and ±75%. An increase in *μ* by 25% and 75% transitions the TFR curve from damped oscillations that reach a steady-state to sustained oscillations (Fig 9A). Decreasing the original value of *μ* by 25% and 75% maintains the qualitative behavior of damped oscillations that reach a steady-state (Fig 9A). Also, there appears to be a Goldilocks value for *μ* which results in the largest cumulative TF (Fig 9B). In contrast, Fig 9C, 9D and 9E show that the qualitative behavior of the TFR does not change as *α*, *β*, or *ζ* are varied by ±25% and ±75%.

In conclusion, variations in *μ* affect the qualitative behavior of the TFR, shifting the TFR curve from damped oscillations to sustained oscillations; thus *μ* may be a bifurcation parameter for a Hopf bifurcation.

#### 3.4.2 Hopf bifurcation with bifurcation parameter *μ*

In order to determine whether *μ* is a bifurcation parameter, we numerically determine the stability of the nondimensionalization of the Fission Model for different values of *μ*. This is done by solving for the eigenvalues through a two step process:

*First*—For each value of *μ*, we numerically solve for the steady-state solutions of the nondimensionalization of the Fission Model using the Multivariate Newton root finding method. The tolerance is set to 0.1x10^−10^ for this method. We compute the steady-state solutions numerically because the analytic steady-state equations involve polynomials of degree ≥26. Thus they are extremely sensitive to numerical error and not ideal for computations [29].

*Second*—We evaluate the Jacobian at the numerically computed steady state values, and then we compute the eigenvalues.

**Fig 10:**
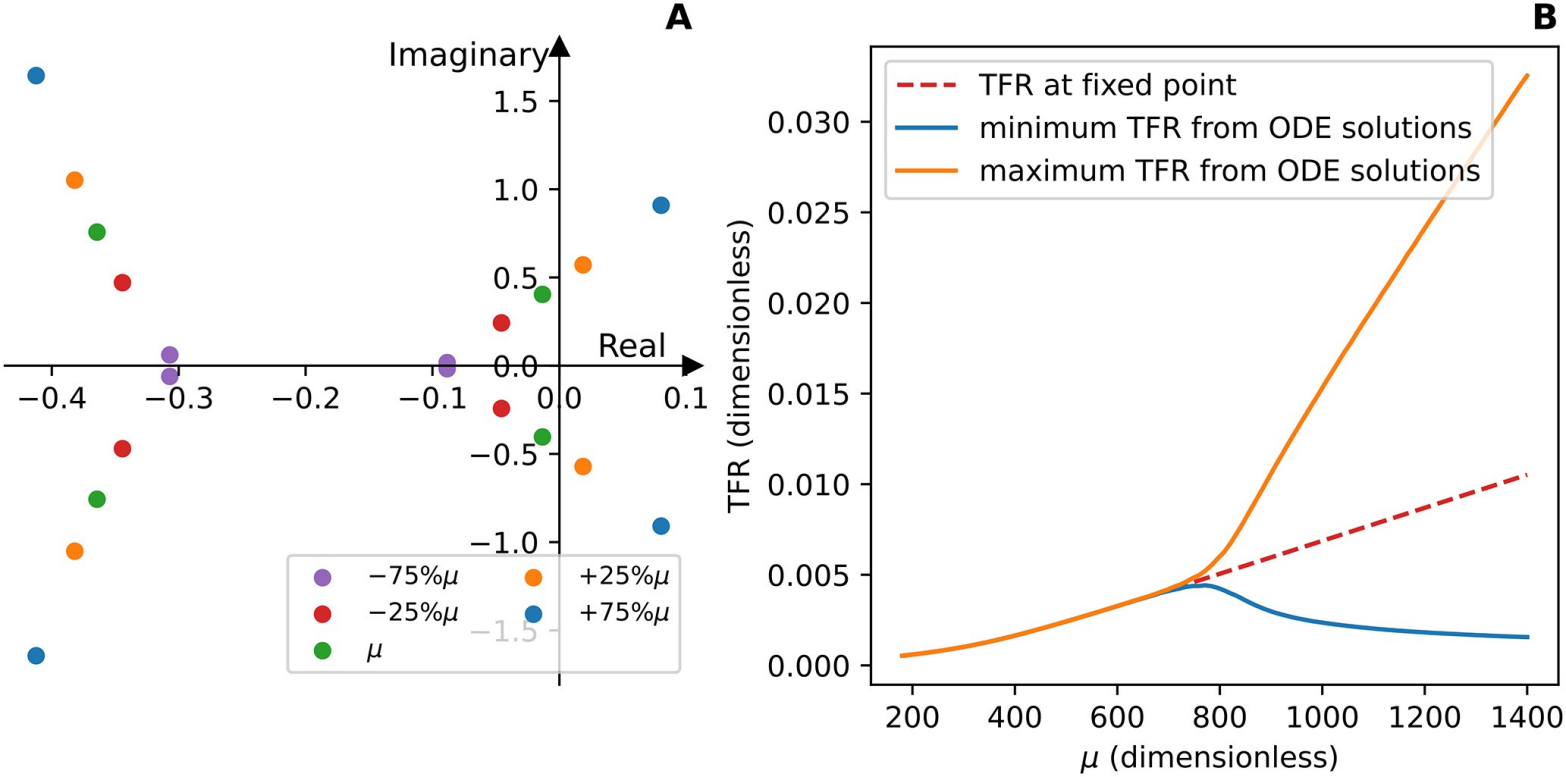
**(A)** shows four of the thirty nonzero eigenvalues from the linearized dimensionless system for each value of *μ* including the eigenvalues with the largest real part. **(B)** is a bifurcation diagram in terms of the dimensionless total fission rate (TFR). The dashed line is the TFR calculated at the numerically computed fixed point for each value of *μ*. The other two curves are the maximum and minimum TFR computed from the numerical solutions of the dimensionless system for each value of *μ*.

Since the nondimensionalization of the Fission Model has two conserved quantities, two of the eigenvalues for each value of *μ* are zero; this leaves 30 nonzero eigenvalues. The four non-zero eigenvalues with the largest real part for each value of *μ* are plotted in Fig 10A. The real part of all 30 eigenvalues for −25% and −75% of *μ* along with the baseline value of *μ* are negative, which indicates the system has a stable fixed point in these three cases. The eigenvalues for + 25% and + 75% of *μ* each have a conjugate pair of eigenvalues with a positive real part. This indicates the system has an unstable fixed point in these two cases. Therefore the TFR undergoes a Hopf bifurcation with bifurcation parameter *μ*. The branching in the bifurcation diagram (Fig 10B) shows the TFR curve transitions from a curve that reaches a steady-state value to a curve with sustained oscillations. The dashed line in Fig 10B is the TFR calculated at the numerically computed fixed point for each value of *μ*. The other two curves are the maximum and minimum TFR computed from the numerical solutions of the dimensionless system for each value of *μ*.

In conclusion, based on numerical evidence, the TFR undergoes a Hopf bifurcation with bifurcation parameter $\mu =\frac{k_{+}M}{k_{-}}$. This indicates that the value of *μ* affects the stability and qualitative behavior of the TFR curve, which leads us to believe *μ* is a key regulatory parameter for the TFR and the cumulative TF. Next we will use Sobol’s method to further quantify how *μ* affects the cumulative TF.

#### 3.4.3 Variance-based GSA for the nondimensionalization

We use Sobol’s method to analyze how each dimensionless parameter affects the cumulative TF. For this *Dimensionless Case*, each variable is assigned a uniform distribution from zero to two times the parameter’s given value. For example, *μ* is assigned the uniform distribution covering (0, 1500) since *μ* = 750. The first and total-order indices (Table 4) show the variation in the cumulative TF is coming almost entirely from *μ*. The first-order and total-order indices are very similar, which indicates there is minimal variation coming from higher order interactions. In fact, we fail to reject the null hypothesis for all second order interactions (Table 5).

**Table 4:** First-order and total-order Sobol indices for the *Dimensionless Case*: The first- and total-order sensitivity indices are calculated from 20, 480 4-tuple samples. First-order indices characterize a parameters individual contribution to the variation in the model’s output. Total-order indices characterize a parameters total contribution to the variation in the model’s output which includes higher order interactions.

*Dimensionless Case*:	*S*_1_	*S*_*T*_
*μ*	0.9966 ± 0.0518	0.9984 ± 0.0480
*α*	0.0000 ± 0.0000	0.0000 ± 0.0000
*β*	−0.0023 ± 0.0040	0.0034 ± 0.0019
*ζ*	0.0000 ± 0.0000	0.0000 ± 0.0000

**Table 5:** Second-order Sobol Indices for the *Dimensionless Case*: The second-order indices were calculated from 20, 480 4-tuple samples. Second-order indices quantify the variation in the model’s output which come from second-order interactions between parameters.

*Dimensionless Case*:	*S*_2_		*S*_2_
*μ* : *α*	0.0008 ± 0.0516	*μ* : *β*	0.0071 ± 0.0531
*μ* : *ζ*	0.0008 ± 0.0516	*α* : *β*	0.0000 ± 0.0000
*α* : *ζ*	0.0000 ± 0.0000	*β* : *ζ*	0.0050 ± 0.0056

**Fig 11:**
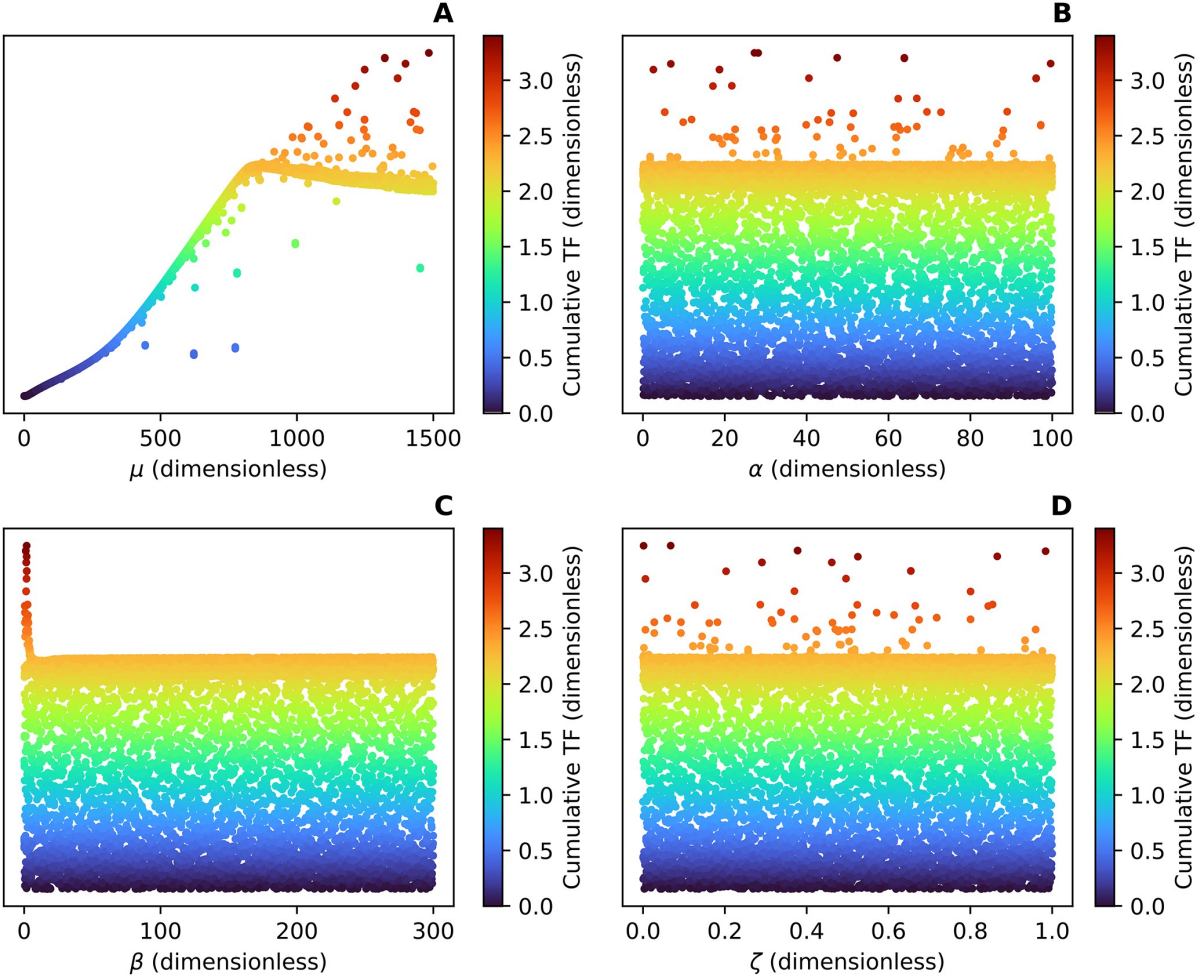
(A), (B), (C), and (D) show the values for *μ*, *α*, *β*, and *ζ* respectively versus the corresponding dimensionless cumulative TF. Note that each point represents a 4-tuple of dimensionless parameter values for (*μ*, *α*, *β*, *ζ*) and the corresponding cumulative TF specific to that 4-tuple.

The Sobol indices in Tables 4 and 5 along with Fig 11A illustrate that the cumulative TF is a nearly perfect function of *μ* and solidify the result that the ratio $\mu =\frac{k_{+}M}{k_{-}}$ is a regulatory parameter for the TFR and the cumulative TF within the Fission Model.

## 4 Discussion

Under extreme metabolic or environmental stress, mitochondria can enter a state of hyperfission which leads to lower functioning, smaller mitochondria and an increase in programmed cell death [7, 8]. However, a total inhibition of fission through deletion of key mitochondrial fission proteins also has negative effects [11]. Thus there exists a need to understand the molecular mechanisms which fine-tune mitochondrial fission to preserve mitochondrial function without deleting key players.

With a nonlinear dynamical system, sensitivity analyses, a nondimensionalization of the system, and a bifurcation analysis we begin to understand how certain parameters affect the total fission rate (TFR) which is the number of fission events per second. These parameters include the association (*k*_1_) and dissociation (*k*_−1_) rates between Drp1 and Mff, the total concentration of tetrameric Drp1 (T) and Mff (M), the association (*k*_+_) and dissociation (*k*_−_) rates between Drp1-Mff oligomers on the OMM, and the fission rate (*f*(*i*)).

Through this investigation, we found the number of fission events per second, the TFR (Eq (15)) depends strongly on *k*_+_, *k*_−_, and M. Individual variations in these three parameters cause the TFR curve to transition from damped oscillations that reach a steady-state to sustained, stable oscillations. Oscillations have been observed in variables associated with mitochondrial dynamics such as ATP production, Ca^2+^ levels, and mitochondrial membrane voltage [30–32]. Our model suggests the TFR as a function of time may oscillate as well. Like the TFR, the cumulative TF, which is the total number of fission events over a set time, depends heavily on *k*_+_, *k*_−_, and M. The largest cumulative TF values occur when *k*_+_ and M are large (Fig 7). For *k*_−_, the range is more nuanced with the largest cumulative TF values occurring when *k*_−_ is between 0.1 and 1 (Fig 7).

Since individual variations in these parameters affected the qualitative behavior of the TFR (Fig 6) and caused the variation seen in the cumulative TF (Tables 2 and 3 and Fig 8), we expected the combination $\mu =\frac{k_{+}M}{k_{-}}$, which can be thought of as the maximum build rate over the disassembling rate of oligomers on the outer mitochondrial membrane, to also have an effect. Indeed, the TFR defined from the nondimensionalization of the Fission Model undergoes a Hopf bifurcation with bifurcation parameter *μ*, implying *μ* determines the stability and qualitative behavior of the TFR. The cumulative TF for the nondimensionalization also depends almost entirely on *μ* suggesting the cumulative TF can be well approximated as a function of *μ* (Tables 2 and 3 and Fig 11A). This indicates there may be a model reduction that only depends on the parameter *μ*.

If we consider $\beta =\frac{k_{1}M}{k_{-}}$ to be less than 5 and fix *μ* to be greater than or equal to the bifurcation value, which is the value of *μ* where the TFR transitions to sustained oscillations, there appears to be another bifurcation with bifurcation parameters *β* and *μ* (Fig 11A and 11C). Future investigations of the Fission Model would elucidate the importance of *μ* and other possible regulatory parameters such as *β*.

The Fission Model represents a well mixed cell with an excess of Drp1 tetramers in the cytosol. This excess in cytosolic Drp1 means the concentration of unbound Mff on the OMM dictates the concentration of *C*_1_ and by extension, the system’s oscillations. Drp1 in excess also means the cytosolic concentration fluctuates minimally over time. Thus the system of equations can be simplified with a quasi-steady-state approximation by setting the equation for $\dot{T}$ (Eq (10)) equal to zero. This quasi steady state approximation would let $k_{1}TM-k_{-1}C_{1}=a\sum_{i=1}^{N}if\left(i\right)C_{i}$. Making this substitution into the equation for $\dot{M}$ (Eq (3)), we see $\dot{M}$ also equals zero in this quasi steady state approximation. This decreases the number of equations from 32 to 30 but unfortunately does not aid in the solving for the steady state solutions. The explicit steady state solutions for the Fission Model are unwieldly and can be found in Leinheiser’s thesis [29].

Since the Fission Model approximates a mechanism with Drp1 in excess in the cytsol, a future direction involves investigating how a smaller pool of cytosolic Drp1 affects the TFR and the cumulative TF. This can be accomplished by decreasing the total concetration of Drp1 and by incorporating other relevant biological phenomenon into the model. The current model returns Drp1 to the cytosol immediately after a fission event. However, Drp1 has been observed to persist on newly created mitochondrial ends for a significant period of time [15]. This delay in the release of Drp1 could be incorporated into the Fission Model with the addition of a delay term or with an additional pool of post fission complexes. Either model addition would affect the available pool of cytosolic Drp1 and possibly cause the total concentration of Drp1 (T) to have more of an effect on the TFR and the cumulative TF.

Another biological phenomenon to consider is the interaction between a mitochondrion and the Endoplasmic Reticulum (ER) before a fission event. Drp1 is know to reside on the outer membrane of the ER [33], and the ER forms close contacts with mitochondria at the eventual fission site marking where fission will occurr [34]. Therefore ER-mitochondria interactions most likely affect the availability of Drp1 for fission activity. The ER also facilitates mitochondrial constriction prior to Drp1 recruitment [34]. A distribution for mitochondrial circumference could be incorporated into the fission function *f*(*i*) which would aid in the understanding of how mitochondrial circumference impacts the number of fission events per second.

Incorporating more complex biological processes like ER-mitochondria interactions would possibly call for association and dissociation rate constants *k*_+_ and *k*_−_ that depend on oligomer size or oligomer location on the OMM. Also, actin filaments and myosin II have been shown to stimulate a maturation process of an oligomer poised for fission to an active fission complex [15]. Presumably *k*_+_ and *k*_−_ should be differet values after this activation step.
